# A New PAMPA Model Proposed on the Basis of a Synthetic Phospholipid Membrane

**DOI:** 10.1371/journal.pone.0116502

**Published:** 2015-02-03

**Authors:** Hui Yu, Qi Wang, Ying Sun, Ming Shen, He Li, Yourong Duan

**Affiliations:** 1 State Key Laboratory of Oncogenes and Related Genes, Shanghai Cancer Institute, Renji Hospital, School of Medicine, Shanghai Jiao Tong University, Shanghai, 200032, China; 2 The Engineering Technology Research Center for Functional Textiles in Higher Education of Guangdong Province, College of Textiles and Clothing, Wuyi University, Jiangmen, Guangdong, 529020, China; 3 Traditional Chinese Medicine Department, Renji Hospital, School of Medicine, Shanghai Jiao Tong University, Shanghai, 200127, China; Griffith University, AUSTRALIA

## Abstract

The purpose of this work was to investigate the synthetic phospholipid dependence of permeability measured by parallel artificial membrane permeability assay (PAMPA) method. Three phospholipids with hydrophobic groups of different lengths and phosphorylcholine as the hydrophilic group were concisely synthesized. Ten model drug molecules were selected because of their distinct human fraction absorbed (%FA) values and various *pK*_a_ characteristics. *In vitro* drug permeation experiments were designed to determine the effect of the incubation time (4–20 h), pH gradient (4.6–9.32) and carbon chain length (8, 10, 12) on the drug permeability through the synthetic phospholipid membrane in the PAMPA system. The results showed that intensive and significant synthetic phospholipids dependence of permeability influenced by the length of lipid’s hydrophobic carbon chain. The effective permeability constant (*P*_e_) of each drug increased rapidly with time, then decreased slightly after reaching the maximum; the pH gradient changed the drug permeability according to the pH-partition hypothesis for drugs with diverse *pK*_a_ values; and longer hydrophobic chains in the synthetic phospholipid membrane improved the drug permeability, as observed for all test drugs at almost all incubation time points. This newly proposed PAMPA model considered the synthetic phospholipid membrane and showed good *P*_e_-%FA correlation for the passive transport of drugs, making it a helpful supplementary method for PAMPA systems.

## Introduction

Because the peroral route for drug administration is the most convenient way to reach systemic therapeutic target sites, the ability of a drug to pass through the gastrointestinal (GIT) barrier is a key property to consider when enhancing the desired beneficial effects of drugs [1]. Several *in vitro* cell-line- (e.g., Caco-2, HT-29, and MDCK) or tissue-based systems are available to assess the potential intestinal permeability of compounds [2–5]. However, these assays are not designed to be performed with high throughput, and high-throughput screening (HTS) is needed to achieve the rapid identification of large numbers of biologically active compounds [6]. Moreover, the permeation mechanisms of these assays are complicated due to their complex constructions [7].

The mechanisms of drug permeation through biological barriers include passive diffusion, active transport, and paracellular and efflux pathways [7]. Recent studies have revealed that 80–95% of commercial drugs are absorbed primarily by passive diffusion [8]. Passive diffusion is a physicochemical process that depends on such underlying physicochemical properties as lipophilicity, hydrogen bonding, *pK*_a_, molecular weight [7] and test conditions, for example, the pH gradient and permeation time [9].

A new high-throughput permeability assay, called the “parallel artificial membrane permeability assay” (PAMPA), was first introduced to investigate passive absorption processes by Kansy in 1998. The PAMPA uses two aqueous buffer solution wells separated by an artificial membrane. The artificial membrane consists of a lipid membrane supported by a porous hydrophobic filter plate matrix. At the beginning of the experiment, the test compound is diluted in buffer and placed in the donor well. The compounds are transferred only by passive diffusion from the donor well into the acceptor well through the artificial membrane. The rate of permeation can be determined by the compound’s effective permeability (*P*_e_) [10]. Because *P*_e_ values are measured and calculated using a UV plate reader, the time required for the PAMPA experiment setup is greatly reduced compared to that required for cell-monolayer methods [11].

Owing to its distinct advantages, considerable studies have extended and improved PAMPA for use in new fields [12]. As well known, there are several factors influencing the PAMPA permeability performance, like unstirred water layer, acceptor and donor solution composition, pH conditions and incubation temperature, et al [1,6]. However, the most important and widely studied experimental parameter is membrane’s lipid composition. Avdeef *et al*. prepared lipid formulations using a 2% w/v n-dodecane solution of highly purified dioleyoylphosphatidylcholine (DOPC), referred to as DOPC-PAMPA [13,14]. DS-PAMPA applied lecithin-based lipid combinations with improved conditions was also described by Avdeef [15]. Sugano *et al*. replicated *in vivo* brush-border membrane conditions using a highly biomimetic phospholipid mixture in a 1,7-octadiene solution (BM-PAMPA) [16–18]. Di *et al*. first reported BBB-PAMPA based on a 2% w/v n-dodecane solution of porcine brain tissue extract and successfully differentiated CNS^+^ from CNS^-^ compounds in 2003 [19], and various PAMPA models were subsequently used to evaluate the passive blood-brain barrier (BBB) permeability of commercial drugs [20–22]. More recently, a measurement method for the transdermal penetration of compounds, termed skin-PAMPA, was also developed [12,23,24]. But, the lipids in these PAMPA models were mainly commercially available lipids or extracted from natural tissues, they had complicated and invariable chemical compositions and, as a result, the most common way to study membrane’s lipid composition was to change the types and proportions of lipids, which limiting our understanding of drug permeation mechanisms in the context of lipid chemical structure.

The aim of our work was to study the significance and extent of membrane’s lipid dependence of permeability from the point of lipid’s chemical structure. Three phospholipid compounds with hydrophobic carbon chains of different lengths were successfully synthesized, and the *in vitro* drug permeability mechanisms of ten model drugs were explored using a synthetic phospholipid membrane in the proposed PAMPA model. The synthetic phospholipids in this study were inexpensive, stable and structurally tailorable, allowing us to explore the effect of the chemical structure of the lipids on the drug permeation mechanism using the PAMPA system.

## Materials and Methods

### Compounds and reagents

2-Chloro-2-oxo-1,3,2-dioxaphospholane (COP) and trimethylamine (TMA) were purchased from Sigma-Aldrich Co. Ltd. (China). 1,8-Octanediol, 1,10-decanediol and 1,12-dodecanediol were purchased from Beckman Co. Ltd (China) and purified by recrystallization before use. α-Methacryloyl chloride (MAC) was synthesized used α-methylacrylic acid and thionyl chloride, purified by distillation, b.p. 95°C [25]. Tetrahydrofuran (THF) was distilled from sodium and stored over a sodium-potassium alloy in a dry box. Triethylamine (Et_3_N) was stirred overnight over KOH and then distilled from CaH_2_. All other reagents were of analytical reagent grade.

The drug compounds used in this study were purchased from the Shanghai Food Drug Analysis Institute (China). PAMPA “sandwiches” were purchased from *p*ION Inc. (USA). Spectroscopic grade dimethylsulfoxide was obtained from Aldrich (China). The pH of the assayed solutions was adjusted with *p*ION buffer solution.

### Synthesis of the phospholipid analog compounds

**Fig 1:**
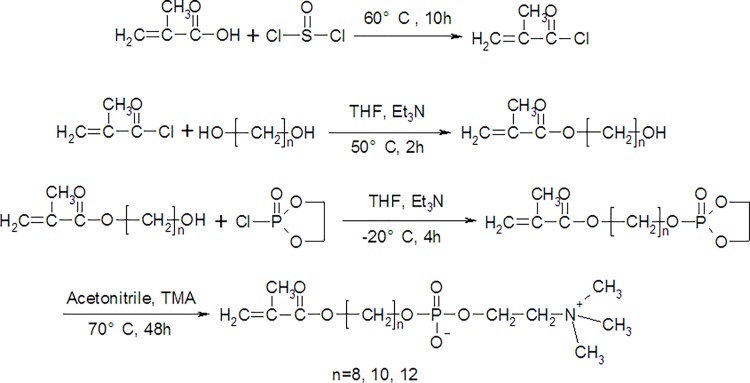
Synthetic scheme for the phospholipids.

The novel family of compounds used in this study was amphiphilic, phospholipid-like compounds containing aliphatic chains as the hydrophobic tail group and phosphorylcholine as the hydrophilic head group. Their synthesis route, according to our previous work, is shown in Fig. 1 [26]. In brief, hydroxyalkyl methacrylate (HAMA) was prepared by reacting various aliphatic chain diols with α-methacryloyl chloride (MAC) at 50°C for 2 h; 2-(2-oxo-1,3,2-dioxaphospholoyloxy)alkyl methacrylate was then prepared by reacting HAMA with COP at -20°C for 4 h; and finally, the ring-opening reaction of 2-(2-oxo-1,3,2-dioxaphospholoyloxy)alkyl methacrylate was performed using trimethylamine (TMA) at 70°C for 48 h, yielding the amphiphilic phospholipid analog compounds. The obtained white hydroscopic powders were dried under vacuum, protected under nitrogen atmosphere and stored at -20°C before use.

### PAMPA assay

The PAMPA evolution instrument from *p*ION Inc. was used in this study. In PAMPA, a “sandwich” structure was formed by a 96-well microtiter plate and a 96-well filter plate from Millipore (IPVH, 125 μm thick filter, 0.45 μm pore), then wetted with a 4 μl 2% w/v phospholipid compound solution in n-dodecane [27].

The stock solutions of the drug samples were prepared at 10 mM concentrations in DMSO and stored at 0°C before use. Before being added to a 96-well filter plate, the stock solution was diluted first with buffer to achieve a final sample concentration of 10∼50 μM and to reduce the DMSO concentration below 1% (v/v).

The PAMPA plate was filled with a 300 μl diluted drug solution to prepare the “donor” wells, and the 96-well filter plate containing a synthetic phospholipid membrane was then placed on the donor wells. The “acceptor” wells were filled with 200 μl of buffer solution and placed on top of the “sandwich”, and the PAMPA instruments were incubated at 25°C in a sealed and saturated humidity container for a specified time. After reaching the permeation time, the PAMPA plate sandwich was separated, and the amount of drugs in both the donor and acceptor compartments were measured by comparing the experimental spectra with the UV spectrum (220∼400 nm) obtained from reference standards.

The *P*_e_ data for the test compounds were calculated using equation [28]:

$$P_{e}=-2.303\frac{V_{a}V_{d}}{\left(V_{a}+V_{d})A(t-t_{0}\right)}\text{lg}\{1-\frac{\left(V_{a}+V_{d})C_{a}(t\right)}{V_{d}SC_{d}(0)}\}$$

where

$$S=\frac{V_{a}C_{a}(t)}{V_{d}C_{d}(0)}+\frac{C_{d}(t)}{C_{d}(0)}$$

V_a_ is the acceptor well volume (i.e., 0.2 ml in this study), V_d_ is the donor well volume (i.e., 0.3 ml in this study), A is the filter area (i.e., 0.2826 cm^2^ in this study), t_0_ is the steady-state time to fill the membrane (i.e., average 1140 s in this study), t is the permeation time, C_a_(t) is the concentration of the drug in the acceptor well at time t, C_d_(t) is the concentration of drug in the donor well at time t, and C_d_(0) is the concentration of the drug in the donor well at the start of the experiment. The factor S was the fraction of samples remaining in the donor and the acceptor wells at time t.

**Fig 2:**
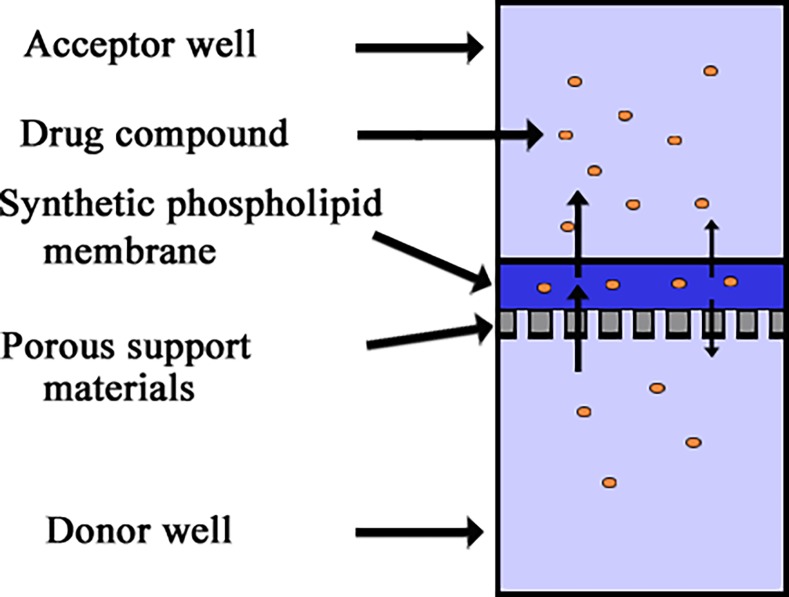
Schematic of the PAMPA model.

The *P*_e_ of each compound was measured at varying incubation times (4–20 h) and pH gradients (4.6–9.32). A schematic of the PAMPA assay is shown in Fig. 2. In the incubation time assays, the pH was 7.4 in both the donor and acceptor wells. In the pH-gradient assays, the pH of the drug solutions was varied using NaOH-treated universal buffer (*p*ION Inc.) in the donor wells, and the pH in the acceptor solution was pH 7.4 [15]. In addition, the incubation time in the pH-gradient test assays was 16 h.

### pH-permeability equation

The pH-permeability equation was based on the pH-partition hypothesis and corrected by taking the effect of the unstirred water layer (UWL) into account [29,30]. For ionizable molecules, there was an equilibrium of un-ionized and ionized species, and the membrane permeation of the dissociable compound was the sum of the permeation of both the un-ionized and ionized species. Based on the pH-partition hypothesis, the following assumptions were made: (1) the permeation of ionized species was negligible; (2) the permeation of un-ionized species occurred through simple passive diffusion across the phospholipid membrane and remained constant over the employed pH range; (3) the UWL permeation also occurred through simple passive diffusion and remained constant over the employed pH range; and (4) the other permeation effectives were negligible. According to these assumptions, the *P*_e_ equation for the monoprotic ionizable molecule was obtained [31].

$$\frac{1}{P_{e}}=\frac{1}{P_{u}}+\frac{\left(10^{\pm (pH-pK_{a})}+1\right)}{P_{o}}$$

Here, ‘+’ denotes acids, and ‘-’ denotes bases. *P*_e_ is the effective permeability, *P*_u_ is the UWL permeability, *P*_o_ is the intrinsic membrane permeability of un-ionized species, and *pK*_a_ is the ionization constant. Hence, it may be suggested that the total resistance to permeation is the sum of the resistances of the membrane and UWL on each side of the membrane [13].

### Statistical analysis

All the drug permeation experiments were performed in triplicates, and the reported values were presented as means ± standard deviation (means±SD). Spearman's correlation coefficient was applied in fitting the correlation between *P*_e_ and the human fraction absorbed, and the fitting data were obtained using commercially available software (Excel, Microsoft office 2010).

## Results and Discussion

### Preparation of phospholipid analog compounds

A series of phospholipid-like compounds containing carbon chains of different lengths were successfully synthesized according to a similar reaction scheme. In brief, one hydroxyl group in the diol molecule was first reacted with MAC (87.2% yield), and then the other hydroxyl group in the diol was reacted with COP (79.6% yield). A ring-opening reaction then gave the targets (84.5% yield). FTIR and NMR confirmed the structures of these phospholipids [28].

### Time assays

**Fig 3:**
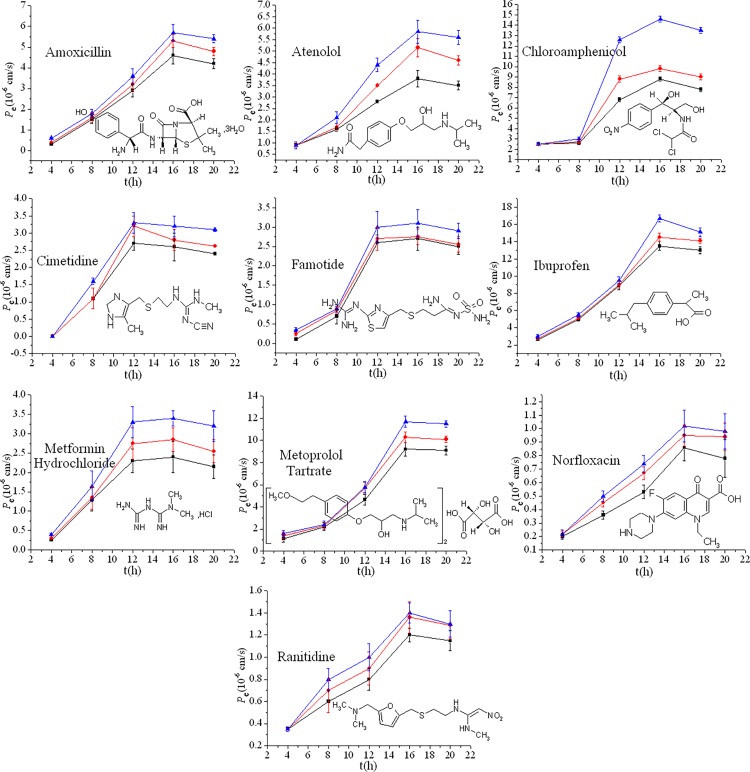
The measured *P*_e_ vs. permeation time for drugs at pH 7.4. The error bars were determined from 3 replicate measurements. Squares denote the phospholipids with octyl chains (C_8_) as the hydrophobic group; dots denote the phospholipids with decyl (C_10_) chains as the hydrophobic group_0_; triangles denote the phospholipids with lauryl (C_12_) chains as the hydrophobic group.

Ten model drugs were chosen in this study to test the synthetic phospholipid membrane PAMPA system. Their physical characteristics, such as molecular weight, solubility, %FA and experimental *P*_e_ data, are listed in Table 1. Fig. 3 presents the measured *P*_e_ values as a function of time for these drugs at pH 7.4. The graphs have a parabola-shaped *P*_e_-t curve. The *P*_e_ initially increased with incubation time and then decreased slightly after reaching a maximum.

**Table 1:** The physical properties and permeability characteristic of model drugs.

Compound	Molecular formula	Mw	Water solubility (mg/)^a^	p*K*_a_^b^	%FA^c^	*P*_e_(10^−6^ cm/s)^d^		
						C_8_	C_10_	C_12_
Amoxicillin	C_16_H_25_N_3_O_8_S	419.46	3430	2.6, 7.31, 9.53	93	4.6±0.4	5.3±0.3	5.7±0.4
Atenolol	C_14_H_22_N_2_O_3_	266.39	1.33×10^4^	9.54	54	3.8±0.4	5.2±0.4	5.9±0.5
Chloroamphenicol	C_11_H_12_C_l2_N_2_O_5_	323.13	2500	4.3	90	8.8±0.2	9.8±0.3	14.6±0.3
Cimetidine	C_10_H_16_N_6_S	252.34	9380	7.23	85	2.6±0.4	2.8±0.3	3.2±0.3
Famotidine	C_8_H_15_N_7_O_2_S_3_	337.45	1000	7.24, 11.19	40	2.7±0.3	2.8±0.2	3.2±0.4
Ibuprofen	C_13_H_18_O_2_	206.28	21	4.59	95	13.9±0.5	14.5±0.5	16.7±0.4
Metformin Hydrochloride	C_4_H_11_N_5_·HCl	165.63	Freely soluble	12.4	55	2.4±0.4	2.9±0.3	3.4±0.2
Metoprolol Tartrate	(C_15_H_25_NO_3_)_2_·C_4_H_6_O_6_	682.82	1.69×10^4^	9.56	95	9.2±0.6	10.3±0.5	11.7±0.5
Norfloxacin	C_15_H_18_N_3_O_3_F	319.34	1.78×10^5^	6.26, 8.63	35	0.9±0.1	1.0±0.1	1.0±0.1
Ranitidine	C_13_H_24_N_4_O_3_S·	314.37	24.7	8.2	55	1.2±0.1	1.4±0.1	1.5±0.1

^a^ Data come from DrugBank database

^b^ Data collected from Chinese Pharmacopoeia, 2005 edition and references [15,27,38]

^c^ %FA data obtained from references [16,40]

^d^ Incubation time is 16h

Based on Fick’s law of diffusion, the permeability was expected to be directly proportional to the buffer-membrane partition coefficient, which was driven by the concentration gradient [15]. In the PAMPA system, the drug first permeated into the phospholipid membrane from the donor well by a passive transport process, and then was transported into the acceptor well from the phospholipid membrane [1]. Due to the high concentration gradient in the incipient incubation process, considerable amounts of drug permeated into the acceptor well from the donor well, and *P*_e_ increased rapidly, as shown in Fig. 3. With further increases in the incubation time, the drug permeability declined in response to the decreased concentration gradient, and an increasing amount of drug was captured within the phospholipid membrane due to hydrogen bonding between the drug and the solvent on the filter [27]. After *P*_e_ reached a maximum, a slight back-diffusion phenomenon occurred, and some drugs returned to the phospholipid membrane from the acceptor well. The *P*_e_ values decreased gradually as a consequence [15].

In summary, Fig. 3 reveals that the different hydrophobic carbon chain lengths of these phospholipids altered the drug permeability; the longer the hydrophobic chain, the better the drug permeation. This was observed at most incubation time points for all the compounds.

### Correlation between *P*_e_ and the human fraction absorbed

Human intestinal absorption (HIA) is a complex process that is involved in many biological events, such as drug dissolution in the gastrointestinal tract and intestinal membrane permeation. The human fraction absorption (%FA) was used to describe the drug intestinal absorption and is defined as the mass ratio of absorbed drug to total drug [32]. In this study, experimental *P*_*e*_ values were compared with theoretical %FA data to investigate the predictability of drug intestinal absorption using the synthetic phospholipid PAMPA system. We selected the data at the incubation time of 16 h as the experimental *P*_*e*_ data because most drugs reach a maximum *P*_*e*_ at this point, and no back-diffusion phenomenon occurred. The correlation between the permeability data obtained from the synthetic phospholipid PAMPA assay and the human absorption data was investigated through Spearman's correlation coefficient. Poor correlations were obtained in the total drug tests, with Spearman's correlation coefficients (R^2^) of 0.745, 0.750 and 0.740 for C_8_, C_10_ and C_12_, respectively, while relatively better correlations (R^2^ = 0.932, 0.948 and 0.955 for C_8_, C_10_ and C_12_, respectively) were received without the three drugs. The three deviated values corresponded to amoxicillin, cimetidine and ranitidine, most likely because they were primarily driven not by passive transport but by carrier-mediated transport and most likely involved a saturable paracellular transport route [33,34]. For the other passively transported drugs, the higher correlations indicated that the proposed method had reliable predictive ability for poorly, moderately and highly absorbed drugs. Moreover, the longer the hydrophobic chain of the synthetic phospholipids, the better the correlation coefficient data for the passive transport of the drugs.

### pH gradient assays

**Fig 4:**
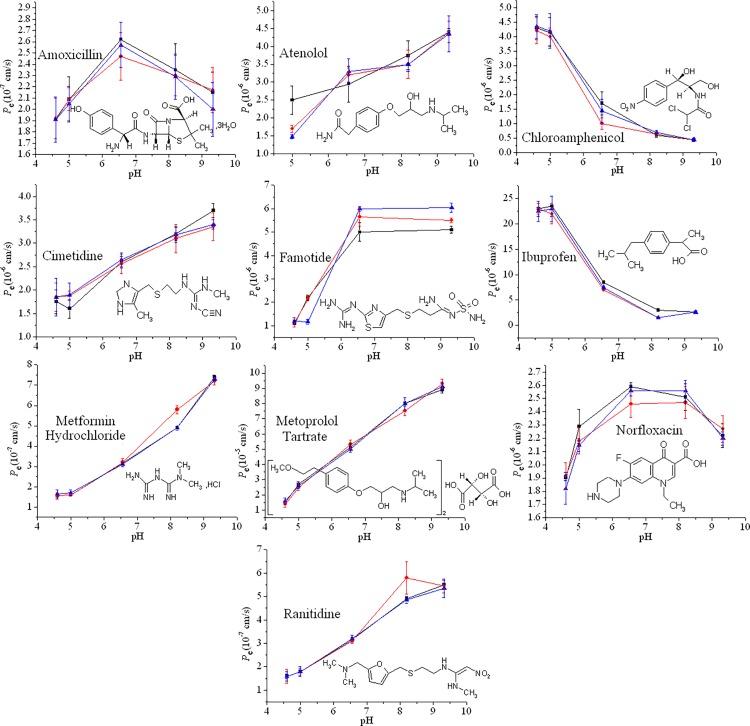
The measured *P*_e_ vs. various pH gradients; the error bars were determined from 3 replicates. Squares denote C_8_, dots denote C_10_, and triangles denote C_12_.

The surfaces adjacent to the enterocytes of the intestinal tract are believed to have an acidic microclimate, ranging from pH 5.2 to 6.0 in the proximal jejunum and to 6.9 in the distal ileum [35,36]. Therefore, many studies have investigated the pH-dependent drug transport mechanism [33,37]. In this study, various pH gradients, ranging from 4.6 to 9.32, were employed in our experiment to determine the pH-gradient effect on the permeation of different drugs across the synthetic phospholipid membranes. Fig. 4 shows the measured *P*_e_ data versus various pH-gradient values (the incubation time was 16 h). Detailed experimental data are provided in Table 2.

**Table 2:** pH-*P*_e_ experimental data.

Compounds	pH	*P*_e_(cm/s)					
		C_8_	S.D.	C_10_	S.D.	C_12_	S.D.
Amoxicillin (×10^–7^)	4.6	1.9	0.2	1.9	0.2	1.9	0.2
	4.98	2.1	0.2	2.1	0.1	2.1	0.1
	6.57	2.6	0.2	2.5	0.2	2.6	0.2
	8.2	2.4	0.2	2.3	0.2	2.3	0.2
	9.32	2.2	0.2	2.2	0.2	2.0	0.2
Atenolol (×10^–6^)	4.6	–	–	–	–	–	–
	4.98	2.5	0.4	1.7	0.1	1.5	0.1
	6.57	3.0	0.5	3.2	0.2	3.3	0.4
	8.2	3.8	0.4	3.5	0.4	3.5	0.2
	9.32	4.4	0.1	4.4	0.3	4.4	0.5
Chloroamphenicol (×10^–6^)	4.6	4.3	0.4	4.2	0.5	4.4	0.4
	4.98	4.2	0.5	4	0.3	4.2	0.6
	6.57	1.7	0.4	1	0.2	1.5	0.4
	8.2	0.6	0.1	0.7	0.1	0.7	0.1
	9.32	0.5	0.1	0.5	0.1	0.5	0.1
Cimetidine (×10^–6^)	4.6	1.8	0.3	1.8	0.3	1.8	0.4
	4.98	1.6	0.2	1.9	0.1	1.9	0.3
	6.57	2.6	0.1	2.6	0.2	2.7	0.2
	8.2	3.2	0.2	3.1	0.3	3.2	0.2
	9.32	3.7	0.2	3.4	0.3	3.4	0.1
Famotide (×10^–6^)	4.6	1.1	0.2	1.2	0.2	1.2	0.2
	4.98	2.2	0.1	2.2	0.1	1.2	0.1
	6.57	5.0	0.4	5.7	0.4	6.0	0.1
	8.2	–	–	–	–	–	–
	9.32	5.1	0.2	5.5	0.1	6.1	0.2
Ibuprofen (×10^–6^)	4.6	23.0	1.5	23.0	1.0	22.5	2.0
	4.98	23.5	2.0	22.0	2.0	23.0	2.5
	6.57	8.5	0.2	7.0	0.1	7.5	0.2
	8.2	3.0	0.1	1.5	0.1	1.5	0.1
	9.32	2.6	0.1	2.6	0.1	2.6	0.1
Metformin Hydrochloride (×10^–7^)	4.6	1.5	0.1	1.5	0.2	1.6	0.2
	4.98	1.6	0.1	1.6	0.1	1.7	0.2
	6.57	3.2	0.1	3.2	0.2	3.1	0.1
	8.2	4.9	0.1	5.8	0.2	4.9	0.1
	9.32	7.4	0.1	7.2	0.2	7.3	0.1
Metoprolol Tartrate (×10^–5^)	4.6	1.5	0.1	1.4	0.2	1.6	0.2
	4.98	2.7	0.2	2.5	0.2	2.6	0.2
	6.57	5.1	0.3	5.3	0.3	5.0	0.2
	8.2	8.0	0.1	7.5	0.3	8.0	0.4
	9.32	8.9	0.2	9.3	0.3	9.1	0.3
Norfloxacin (×10^–6^)	4.6	1.9	0.0	1.9	0.1	1.8	0.1
	4.98	2.3	0.1	2.2	0.1	2.2	0.1
	6.57	2.6	0.0	2.5	0.1	2.6	0.0
	8.2	2.5	0.1	2.5	0.1	2.6	0.1
	9.32	2.2	0.1	2.3	0.1	2.2	0.1
Ranitidine (×10^–7^)	4.6	1.6	0.1	1.6	0.3	1.6	0.2
	4.98	1.8	0.2	1.8	0.2	1.8	0.2
	6.57	3.2	0.1	3.1	0.1	3.2	0.2
	8.2	4.9	0.1	5.8	0.7	4.9	0.2
	9.32	5.5	0.2	5.5	0.3	5.4	0.4

Walter and Gutknecht [29] and Avdeef [13] proposed a pH-permeability equation based on the pH-partition hypothesis corrected for the effect of the unstirred water layer (UWL). In this equation, the membrane permeation of the ionized form of the molecule was negligible, the total resistance to permeation was the sum of the resistances of the membrane and the UWL on each side of the membrane [18], and the effective permeability (*P*_e_) was determined by the membrane permeability (*P*_m_) and the unstirred water layer permeability (*P*_u_).

The UWL resistance was rate limiting for highly permeable molecules. For a weakly acidic drug, the effect of the UWL was to lower the *P*_e_ in acidic conditions [14]. If the UWL was absent, *P*_e_ took the intrinsic value, *P*_o_. The concentration of the unionized drug decreased by an order of magnitude for every unit increase in the pH based on the pH-partition hypothesis [30], as demonstrated by the curves for chloramphenicol and ibuprofen, which had single low *pK*_a_ values, as listed in Table 1. For a weakly basic drug, the effect of the UWL was to lower the *P*_e_ in basic conditions. If the UWL was absent, the concentration of the unionized drug increased for every unit increase in the pH according to pH-partition hypothesis [38], as the curves for atenolol, cimetidine, famotidine, metformin hydrochloride, ranitidine and metoprolol tartrate show in Fig. 4. Furthermore, as the permeability decreased, the measured errors in Fig. 4 increased due to the reduction of the drug concentration in the acceptor well and the back-diffusion phenomenon [39].

Only two drugs in Fig. 4, amoxicillin and norfloxacin, were expected to exist in a zwitterionic form because of their weak acid and base groups with reported different *pK*_a_ values, resulting in hyperbolic-shaped *P*_e_-pH curves as shown in Fig. 4, with the maximum point in the hyperbolic permeability curve near pH 7.0 [15].

For the various phospholipid membranes, Fig. 4 reveals that the different hydrophobic chains have no special effect on the permeation of these diverse drugs.

## Conclusions

To study drug permeability influenced by synthetic phospholipid membrane in the PAMPA system, three amphiphilic phospholipids were successfully synthesized, and ten drugs were selected as model drugs. The properties of drug permeation were investigated by varying incubation times (4–20 h), pH gradients (4.6–9.32) and using different synthetic phospholipids with hydrophobic chains of different lengths.

Our data showed a significant effect of synthetic phospholipid membrane on drug permeability. Upon permeating, *P*_e_ increased rapidly with time, but decreased slightly after reaching a maximum. The pH gradient experiments showed that the modeling of the different pH values in human absorption was fairly accurate, as the pH-partition hypothesis was able to determine the drug permeabilities. Lengthening the hydrophobic chain of the synthetic phospholipids increased both the drug permeation and the accuracy of the experimental data compared with %Fa values.

Our PAMPA assay utilized synthetic phospholipids and could be a complementary tool for the PAMPA system. Our assay can facilitate a better understanding of the drug permeation mechanism in PAMPA through the application of various simple synthetic phospholipids and broaden PAMPA’s application by employing different types of phospholipid compounds. However, to predict different tissue permeation needs, more biologically analogous synthetic phospholipids are needed to mimic the tissue membrane layer; this further work is currently underway.
